# Penn, Social Systems, and the Community: fostering equity-focused public health practice through asynchronous learning

**DOI:** 10.3389/fpubh.2026.1748515

**Published:** 2026-02-11

**Authors:** Anita Stief, Elaine Weigelt, Kaliya Greenidge, Nekia Rosado, Moriah Hall, Jaya Aysola, Hillary C. M. Nelson

**Affiliations:** 1 Master of Public Health Program, Perelman School of Medicine, University of Pennsylvania, Philadelphia, PA, United States; 2 Center for Health Equity Advancement, University of Pennsylvania, Philadelphia, PA, United States; 3 Penn Center for Public Health, University of Pennsylvania, Philadelphia, PA, United States; 4 Division of General Internal Medicine, Department of Medicine, Penn Medicine, Philadelphia, PA, United States; 5 Leonard Davis Institute of Health Economics, University of Pennsylvania, Philadelphia, PA, United States; 6 Department of Biochemistry and Biophysics, Perelman School of Medicine, University of Pennsylvania, Philadelphia, PA, United States; 7 Family Medicine & Community Health, Perelman School of Medicine, University of Pennsylvania, Philadelphia, PA, United States; 8 Department of Pathobiology, School of Veterinary Medicine, University of Pennsylvania, Philadelphia, PA, United States

**Keywords:** anti-racism, applied practice experience, asynchronous learning, graduate education, health equity, pedagogy, public health education, structural racism

## Abstract

Penn, Social Systems, and the Community (PSSTC) is a semester-long, non-credit, asynchronous course designed to prepare the University of Pennsylvania (UPenn) Master of Public Health (MPH) students for community engagement by analyzing how historical and systemic inequities impact public health. As a prerequisite for the Applied Practice Experience (APE), PSSTC addresses a key pedagogical challenge in public health education: providing foundational training on structural inequalities and their influence on public health practice. The curriculum includes nine online modules and four synchronous discussions covering topics such as racism and other forms of oppression, social determinants of health, implicit bias and microaggressions, transformative justice, and the role of UPenn itself in these broader systems. A post-course survey was administered and respondents agreed that the course prepared them for the applied practice experience and other public health work. Students also reported gaining knowledge and practical strategies for working with diverse populations. Suggested improvements included condensing content for more focused learning and incorporating broader perspectives from underrepresented racial, ethnic, and religious populations. In response, course revisions are ongoing to streamline content and ensure alignment with the evolving public health landscape.

## 1 Introduction

For decades, racial scholars have documented that racism drives disparities in morbidity, mortality, and overall well-being based on socially assigned race. Structural racism is embedded within, and reinforced by, multiple societal systems, including housing, employment, credit lending, education, the criminal justice system, the economy, and healthcare. Importantly, racism adapts over time, maintaining its pervasive effects through new mechanisms that emerge as older forms weaken (1, 2).

The events of 2020, including the COVID-19 pandemic and the nationwide racial uprising following the murder of George Floyd, highlighted these longstanding issues. A systematic review and meta-analysis found that African American, Hispanic, and Asian American individuals were at considerably higher risk of COVID-19 positivity and ICU admission compared to White individuals. Furthermore, socioeconomic disparity and clinical care quality were strongly associated with COVID-19 mortality outcomes in racial and ethnic minority groups (3). Concurrently, the resurgence of the Black Lives Matter movement amplified public discourse around equity, diversity, and inclusion (EDI) (4). The convergence of these events reinforced what public health scholarship has long established: racism is a public health crisis. They also underscored the urgent need to equip future public health professionals to respond effectively to such challenges. Consequently, training in cultural competence should be intentionally integrated into institutional curricula (5).

While cultural competence is a skill that can be taught, trained, and achieved, the concept of cultural humility deemphasizes cultural knowledge, placing greater emphasis on a lifelong process of self-evaluation and critique, and promoting interpersonal sensitivity and addressing power imbalances (6). The Council on Education for Public Health (CEPH) 2024 accreditation criteria further require programs to advance diversity and cultural humility. CEPH defines cultural humility as the practice of bridging cultural gaps between public health professionals and communities to promote culturally sensitive and population-centric strategies. To practice cultural humility, public health professionals must be aware of their own cultural biases, acquire knowledge about different cultures, and demonstrate respect and sensitivity to diverse cultural perspectives (7).

However, these frameworks often fall short of directly addressing racism as a structural determinant of health. Historically, training programs for health professionals have identified cultural competence as a core curriculum objective. Over time, cultural competence education in some academic settings has tended to focus on the power and privilege associated with whiteness (8). Similarly, some programs have moved away from the gentler concept of “multicultural education” toward the more challenging and necessary framework of “anti-racist education” (8). Increasingly, racism is being recognized as a problem with deep, intergenerational effects, that requires systems change, flexibility, and structural power analysis to confront effectively (9).

While cultural competence and humility are important, it is equally crucial that students engage early in conversations about structural racism and its impact on health outcomes. Conversations about race at the outset of an academic program allow students to confront the ways structural racism produces disparate health and education outcomes and launch them with the language for discussing how to mitigate them (10). Although academics have chronicled the role of racism in shaping health outcomes, we have not examined how our own schools and programs of public health perpetuate racism. Furthermore, we have largely failed to prepare our graduates with an understanding of the roots of racism and how it affects public health work (8). Additionally, academic departments rarely discuss equity, diversity, and inclusion (EDI), and when they do, they often highlight the need for increased physical diversity. Graduate programs need to make greater efforts to engage students’ socially constructed identities and integrate EDI concepts into curriculum, co-curricular, and field experiences to better prepare students to address societal problems (11).

This paper examines a response to this pedagogical challenge by analyzing the implementation of a novel anti-racism curriculum within the University of Pennsylvania’s (UPenn) Master of Public Health (MPH) program. Specifically, we analyzed the perceived effectiveness of asynchronous modules in preparing MPH students for the Applied Practice Experience (APE), which was formerly referred to in our program as fieldwork, and other areas of public health practice. The findings of this study are exploratory and may offer a replicable model for integrating anti-racism education into public health training, ultimately strengthening graduates’ capacity to work effectively anywhere in the world and with diverse populations (7).

## 2 Pedagogical framework

In the Summer of 2020, the UPenn MPH program convened listening sessions with current students and alumni to provide a safe space for reflection and dialogue. These sessions sparked the creation of a student-led organization, the Racial Equity Student Advisory Council (RESAC). The goal of RESAC was to provide a supportive space for connection, dialogue, and action around racial justice in public health. RESAC submitted a letter to MPH program leadership which outlined specific demands for anti-racism support systems and the need for policy and practice changes within the MPH program and the broader Perelman School of Medicine. A key theme that emerged from the letter and listening sessions, specifically from Black and Brown students, was the need to integrate anti-racism education into the MPH curriculum. Students emphasized that this was critical to engage with and work alongside diverse communities equitably.

To address these student and alumni concerns, the MPH program leadership began exploring options for integrating anti-racism education into the MPH curriculum. Adding a formal credit-bearing course to the curriculum would add tuition cost to our learners and pushed us to think creatively about how to integrate this important content. Instead, we leveraged a new online, asynchronous course, *The Penn Experience: Racism, Reconciliation, and Engagement*, which was co-developed in 2019 by two faculty members from the University of Pennsylvania’s School of Social Policy and Practice (SP2) and the Penn School of Dental Medicine (PDM) (12, 13). They had developed this course in response to feedback from students across both schools, especially students of color, that their classmates were not sufficiently prepared for classroom discussions about oppression or interactions across differences in clinical spaces. The course was designed to maximize flexibility and allow the content, timing, and grading system to be tailored to students across schools and programs during the COVID-19 pandemic, when learning was taking place largely online (12, 13).

Because of our strong partnerships with SP2 and PDM, reflected in both the enrollment of dual degree students across these schools and the shared faculty who teach in multiple programs, the MPH program was able to access their core curriculum. However, given the clinical nature of SP2 and PDM training, we needed to reimagine portions of the curriculum to make it more appropriate for public health students. A small group of Penn faculty and staff adapted existing content and created new material, resulting in an innovative, asynchronous, non-credit-bearing course, renamed *Penn, Social Systems, and the Community* (PSSTC). The revised course emphasizes Penn and Philadelphia, public health competencies, and supporting communities.

Although an overarching theoretical framework for the course was not formally laid out, the course goals and content followed the pedagogical principles and the core tenets of Critical Race Theory, which fundamentally posits that racism is endemic and, although race is a social construct, it has significant impacts on people’s everyday lives. The curriculum also includes other theoretical frameworks for understanding racism and oppression, such as intersectionality, settler colonialism, and post-colonial theory (12, 13).

The learning goals for PSSTC are to prepare students to critically analyze the impact of historical and contemporary social, economic, political, and institutional inequities on community health outcomes with a focus on Philadelphia. The curriculum provides important content not always covered in the MPH core courses, aiming to help students connect classroom learning to real-world public health issues and foster discussion among peers. By the end of the course, students should gain essential frameworks and knowledge to critically assess systemic factors and advance equitable public health practices, laying a solid foundation for their APE and future careers in public health.

## 3 Learning environment

The University of Pennsylvania’s Master of Public Health (MPH) is committed to fostering an intentionally diverse learning environment. The program welcomes students of varied races, ethnicities, ages, public health interests, and professional backgrounds, including clinical specialties (medicine, dental, veterinary, nursing), social work, law, and more. The field of public health transcends sectors, disciplines, and communities, and it is essential that the MPH program reflects the multifaceted nature of society. This diversity enriches the learning environment by exposing students to varying perspectives and lived experiences, preparing them to work effectively across fields and with communities from all backgrounds.

According to data reported by UPenn to the Association for Schools of Public Health (ASPPH) during the 2024 reporting cycle, the racial and ethnic composition of the MPH student body was as follows: 38.3% self-identified as Non-Hispanic White, 22.7% as Asian, 21.3% as Black/African American, 11.3% as Two or More Races, 4.3% as Hispanic/ Latino, and 2.1% selected Unknown Race. Data for Pacific Islander students was not collected or reported separately at the time of this 2024 data submission. Females comprised 79% of enrolled students, males 18%, and nonbinary students 3%. International students accounted for 6% of the overall study body. During the same reporting cycle, there were 36 faculty members holding titles such as Assistant Professor, Associate Professor, Professor, Lecturer, or Other. Over was 80% of faculty self-identified as Non-Hispanic White and over 11% Black-African American, about 3% Hispanic/Latino, about 3% Asian, and about 3% identified as two or more races. Faculty gender distribution was 75% female and 25% male.

PSSTC is a prerequisite to the MPH Applied Practice Experience (APE), a practice-based requirement in which students apply core public competencies within real-world settings aligned with their interests. The APE requires a minimum of 125 hours of supervised work and is designed to strengthen students’ capacity to engage in meaningful public health practice. The integration of PSSTC ensures that students enter APE with historical knowledge, skills, and tools necessary to collaborate effectively with communities, recognizing the various social determinants of health that impact populations.

Directed by a dedicated MPH faculty member and supported by a student teaching assistant (TA), the instructional team guides students through the curriculum, facilitates engagement with asynchronous modules, and supports collaborative learning. The course runs every semester, in fall, spring, and summer, with an average class size of 16 students (range: 10–23), depending on the semester. Students take PSSTC early in their MPH training, typically in the first semester for full-time students and the first or second semester for part-time students. Given the nature of the course content, faculty receive professional development opportunities, including workshops on restorative facilitation, conflict styles and communication, developing emotional intelligence, and navigating difficult conversations. Faculty then support teaching assistants in managing emotionally charged discussions.

The current iteration has nine modules. The first seven modules give a background on Philadelphia, an introduction to racism and other forms of oppression, the relationship and responsibility of the University of Pennsylvania to its surrounding neighborhoods, an overview of the social determinants of health, an introduction to health and healthcare, a discussion of environmental racism, and lessons on implicit bias, microaggressions, and imposter syndrome. The eighth module focuses on personal responsibility, giving students a choice of material, while the last module focuses on providing tools to move forward and take action. Students are expected to authentically engage with the module content, spending approximately 2 hours per module. Those familiar with the content are encouraged to explore the additional resources provided to deepen their understanding.

At the end of each module, students complete a short reflection activity. These activities fostered critical thinking and encouraged students to explore the connection between their personal identity, lived experiences, and public health practice. For example, students used a wheel diagram to map experiences of advantages or disadvantages across identity categories and explored how they related to their public health interests and passions. In another activity, students used the Southeastern Pennsylvania Regional Community Health Needs Assessment (rCHNA), which assesses population health and social needs indicators, to map a specific health measure and identify nearby community assets. Reflections were reviewed by the course director, who provides individualized feedback.

Several times a semester students engaged in a synchronous Zoom session to create a hybrid learning environment that promotes flexibility and accessibility and a learner-centered pedagogy. This was especially important since many of our students are part-time learners. These meetings provide a safe space for real-time discussion, peer learning, and direct Q&A. During sessions, students engage in small and large group discussions, learn about their classmates’ APE experiences, hear from APE partners about their organizations, and learn from alumni working in public health.

At the conclusion of the course, an anonymous post-course Qualtrics survey is distributed via email. The survey is designed to collect feedback for quality improvement and to inform future iterations of the course. It also allows us to assess students’ preparedness to engage with Philadelphia and the surrounding communities during their APE experience and in future public health practice. Reminders to complete the survey were sent through the Canvas announcements function.

## 4 Approach and results

### 4.1 Study aim

The aim of this study was to use the responses from the post-course survey to evaluate the perceived effectiveness of the asynchronous modules in preparing MPH students for APE and other areas of public health practice. This study looked at data from five academic semesters (Summer 2023, Fall 2023, Spring 2024, Summer 2024, and Fall 2024). During the study period, the APE was known as the fieldwork experience, a terminology reflected in both the survey statements and the resulting quantitative and qualitative data. In this paper we focus on the asynchronous modules and the overall impact of the course on the students’ learning.

### 4.2 Processes and tools

Following the end of the PSSTC course, students were asked to complete a survey where they rated the degree to which they agreed with the following statement: “The following asynchronous content in this module has helped prepare me for my fieldwork and/or other public health practice.” In addition to a question for each module, students were also asked whether the course improved their understanding of Philadelphia and the University of Pennsylvania, as well as whether the amount of content in the course felt manageable (an important question, given that this course was non-credit bearing). The survey used a 5-point Likert scale where 1 indicates “Strongly Disagree,” 2 indicates “Somewhat Disagree,” 3 indicates “Neither Agree nor Disagree,” 4 indicates “Somewhat Agree,” and 5 indicates “Strongly Agree.” Due to the small sample size, the analysis was confined to descriptive statistics, with agreement defined as the proportion of students who selected Somewhat Agree or Strongly Agree to each Likert scale question.

In addition to Likert-scale questions, the Qualtrics survey included five open-ended questions that probed general perceptions of the overall course experience to assess how effective the asynchronous modules were in preparing MPH students for fieldwork and other public health practice. Of the total 79 participants over five academic semesters, we had a total of 23 respondents, for a rate of 29%. The data collected over the five academic semesters and analysis are reflective of the iteration of PSSTC at the time. Given the small sample size and formative purpose of the evaluation, the quantitative analyses were limited to descriptive statistics.

Across all 12 questions, agreement rates (defined as somewhat agree or strongly agree) ranged from 57.89% to 100.00% (see Table 1). Nine out of 12 Likert scale questions had agreement rates of 90% or higher, demonstrating an overall strong perceived effectiveness of the asynchronous modules (see Table 1). The high-scoring questions included 7 of the modules, as well as general questions about the usefulness of the course in preparing students for fieldwork. The highest scoring module was the one on *Social Determinants of Health (Impacts on Health Outcomes),* with an agreement rate of 100.00% (see Table 1). In addition to the social determinants of health, this module focused on racism and its impact on health, environmental racism, and the social and built environment. The high level of agreement suggests that this module impacted perceived preparedness for the fieldwork experience and may become a foundational component in future course iterations.

**Table 1:** Descriptive statistics of Likert-scale responses.

Statement	Agree #	Total #	% agree
The asynchronous content in Module 1 (course framing: racism and other forms of oppression) has helped prepare me for my fieldwork and/or other public health practice	20	22	90.91%
The asynchronous content in Module 2 (welcome to Philadelphia) has helped prepare me for my fieldwork and/or other public health practice	21	23	91.30%
The asynchronous content in Module 3 (Penn’s responsibility) has helped prepare me for my fieldwork and/or other public health practice	20	22	90.91%
The asynchronous content in Module 4 [social determinants of health (impacts on health outcomes)] has helped prepare me for my fieldwork and/or other public health practice	22	22	100.00%
The asynchronous content in Module 5 (health and healthcare) has helped prepare me for my fieldwork and/or other public health practice	21	22	95.45%
The asynchronous content in Module 6 (the relationship between genetics and environment) has helped prepare me for my fieldwork and/or other public health practice	20	22	90.91%
The asynchronous content in Module 7 (implicit bias and microaggressions) has helped prepare me for my fieldwork and/or other public health practice	17	19	89.47%
The asynchronous content in Module 8a (personal responsibility-for students that identify as white) has helped prepare me for my fieldwork and/or other public health practice	11	19	57.89%
The asynchronous content in Module 8b (personal responsibility-for students that identify as black, indigenous or as people of color) has helped prepare me for my fieldwork and/or other public health practice	12	18	66.67%
The asynchronous content in Module 9 (moving forward, taking action) has helped prepare me for my fieldwork and/or other public health practice	17	19	89.47%
This Canvas course has improved my understanding of Philadelphia & Penn	21	22	95.45%
The amount of content in this canvas course felt manageable	21	23	91.30%

Respondents rated their agreement with the following statements, using a 5-point Likert scale, with agreement defined as somewhat agree or strongly agree.

While most modules had a high agreement rate, the lowest levels of agreement were found for the two modules on Personal Responsibility. One of these modules covered topics such as the history of racial construction, White supremacy, White fragility, the White savior complex, and allyship; this module was recommended for students who identify as White, and it had an agreement rate of 58% (see Table 1). The other Personal Responsibility module covered topics such as racial literacy, racialized violence, intergenerational trauma, imposter syndrome, and internalized racism; this module was recommended to students who identify as Black, Indigenous, or People of Color (BIPOC), and it had an agreement rate of 67% (see Table 1).

Overall, the results indicate that although students who responded to the post-course survey generally agreed that the asynchronous modules helped them prepare for fieldwork and other aspects of public health practice, the “Personal Responsibility” modules require further review. Specifically, the data highlights the complexity of integrating racial identity into public health education. This presents an opportunity to gather more specific feedback from students to help identify sources of discomfort, resistance, or perceived misalignment. Further questions should be designed with careful attention to ethical and pedagogical sensitivity, recognizing the evolving social and educational context in which students engage with identity, responsibility, and anti-racist content. The goal is to revisit and refine this content to ensure it is inclusive, supportive, and relevant to the diverse experiences of both MPH students and the Philadelphia community.

### 4.3 Qualitative analysis of survey responses

Of the 23 students who completed the survey, 21 students responded to at least one of the five qualitative questions: (1) Could you elaborate and provide examples of what asynchronous you would like to see modified; (2) Could you explain why you liked the content as-is?; (3) Please describe what your takeaways were from this course; (4) Can you provide some constructive feedback on what would make this course more valuable to you?; (5) Please provide any additional comments you have on the course overall.

Free-response items on the post-course survey were reviewed iteratively and analyzed inductively to identify recurring patterns. Responses were grouped into thematic categories and refined through repeated comparison using Microsoft Excel. Given the small sample size, the analysis was intended to provide descriptive insights into the course’s strengths and areas for improvement rather than a formal qualitative analysis. The responses were organized into five thematic categories: Application, Content, Gratitude, Lifelong Learning, and Logistical.

### 4.4 Application

Students commented not only on the knowledge gained but also on the practical strategies they could implement in the field. Many students valued the perspectives offered in the modules and noted how learning about these issues is critical before beginning fieldwork. One student reflected: *“Knowledge + Practical strategies to employ in the field especially when working with specific populations. The importance of being self-aware when it comes to biases and micro aggressions, how to stand up for others and not just yourself while being respectful. Why it’s necessary to approach care from a holistic standpoint instead of making assumptions and being narrow-minded. Addressing social determinants of health as part of standard patient and population care.”* These comments demonstrate alignment with the course’s purpose and goals.

### 4.5 Content

Several students mentioned specific modules they found valuable and suggested additional content. For instance, a student noted: *“(1) an overview of Philly history; (2) the history of healthcare and public health; (3) the history of Penn; (4) and the* var*ious-isms that interplay with people’s experiences in Philly, at Penn, and within the context of public health and healthcare.” Another student commented: “Different perspectives/sides on some of the more controversial issues here. Would like to better understand some of the more thoughtful but contrarian perspectives on Health/Healthcare; Genetics/Environment; Affirmative Action.”* These comments reflect both appreciation for existing material and a desire for more diverse viewpoints in future iterations.

### 4.6 Gratitude

Students expressed overwhelmingly positive sentiments about the asynchronous course, finding it valuable for both their personal lives and professional careers. They described the experience as enjoyable and appreciated having access to the content prior to beginning their fieldwork. One student shared: *“Thank you for putting the work into this course! This is a bundled breakdown of important topics that I was comfortable with but needed to dive deeper into. I like the idea of passing something like this collection of materials onto family members and colleagues.”* This theme highlights that students not only resonated with the materials but also appreciate the flexibility and structure of the asynchronous learning format.

### 4.7 Lifelong learning

Students noted the importance of ongoing education around the course topics. Their comments emphasized the need to unlearn certain behaviors, recognize privilege, and learn about communities beyond the University of Pennsylvania bubble. One student reflected: *“I really took away the idea that as a white, straight woman in this world, I constantly need to be relearning and unlearning and be open to doing so. Being aware of my privilege is so important, and it’s my job to help others see and understand their privilege in society as well.”* The theme underscores students’ recognition that continued education is essential for supporting communities in public health practice.

### 4.8 Logistical

Students offered feedback on how to enhance the course’s accessibility and organization. Suggestions ranged from incorporating more diverse perspectives to condensing the material. For example, one student commented: *“A LOT of readings/background content. If this could be condensed, that would be very helpful.”* Another student offered feedback, noting *“The content is designed in such a way that it flows together, and each module builds on one another, which makes what we learn easier to grasp and understand.”* These comments highlight both tangible opportunities for course revision and strengths in the current design that support student learning and engagement.

The five identified themes demonstrate the value of gathering student feedback to gain insight into the strengths and opportunities for improvement within the asynchronous modules. Overall, students valued the knowledge and practical strategies gained, and several commented on the importance of this content before starting their APE. They also highlighted the importance of ongoing learning and reflection in preparing for public health practice.

## 5 Discussion

Master of Public Health (MPH) students who engaged with Penn, Social Systems, and the Community (PSSTC) between Summer 2023 and Fall 2024 and responded to the post-course survey generally agreed that the asynchronous modules helped them prepare for APE and other aspects of public health practice. The lowest agreement rates were observed for the modules on personal responsibility, which segmented students based on how they identified, either as White or as Black, Indigenous, or a Person of Color. However, no qualitative comments directly addressed these modules, which highlights an opportunity to revise the Qualtrics survey to elicit module-specific qualitative feedback.

Future iterations must also consider the literature on anti-racist pedagogy, which emphasizes that the journey toward cultural humility will look different for students from different backgrounds (14). Furthermore, the research highlights the importance of decentering Whiteness, even within antiracist education, as students of color can face “racial battle fatigue” from having to listen to their white counterparts discover, confess, and have their emotions take center stage (14). On the other hand, when educators engage white students in race conversations, they may perceive it as a personal attack and grow defensive (10). This possible challenge between engaging white students thoughtfully and preventing “racial battle fatigue” for students of color highlights the need for this course to be facilitated by faculty who are equipped to navigate these conversations (11). This finding also underscores an opportunity not assessed in this study: training faculty alongside students. Faculty should understand what students are learning and be prepared to use these frameworks to guide classroom discussions around anti-racist pedagogy. Incorporating faculty development will be essential for fostering thoughtful, effective, and inclusive dialogue.

Student feedback from these surveys informed significant revisions to the course’s format and scope. For example, one student wondered if the unique lived experiences of additional ethnic and racial groups could be added. Furthermore, several comments suggested that there was a lot of content and background information, and it would be helpful and less overwhelming if it were condensed. In response to this feedback, we continue to iterate on the course content to incorporate feedback on expanding cultural, historical, sociopolitical, and religious perspectives. Additionally, the nine modules have now been pared down to “five sections” to consolidate the course content and address the feedback about the overwhelming nature of the course and the need to lessen the content. This condensed approach eliminated the choice of completing only one of the personal responsibility modules, and students are now encouraged to move through the content together.

The program’s commitment to integrating student perspectives is also evident from a student who identified a gap in PSSTC content on immigrant health and resilience. The program supported her in developing a project that included a landscape analysis of the literature, a focus group with peers and community members, and the creation of section content.

The findings from this evaluation underscore the critical need for more research on effective anti-racism education in public health. While the Council on Education for Public Health (CEPH) requires schools and programs of public health to “discuss the means by which structural bias, social inequities and racism undermine health and create challenges to achieving health equity at organizational, community and systemic levels” (7), few peer-reviewed studies provide guidance on how to do so effectively. A systematic review identified 11 examples of teaching about racism, but only seven reported any evaluation of outcomes. These findings suggest that consensus remains limited on best practices for teaching antiracism (15).

Our evaluation contributes to this evidence base and adds to the conversation on how to prepare public health students for anti-racist practice. PSSTC can serve as a model for other MPH programs, as most of the module content is transferable and the Welcome to Philadelphia and Penn’s Responsibility modules can be tailored to align with the specific needs of any city and institution. Interested MPH programs should examine their university’s policies, processes, and institutional culture for supporting an initiative like this. Moreover, faculty and staff support is necessary for updating content, grading module activities, and engaging with students through synchronous sessions (16). To sustain these efforts, programs must think about faculty allocations or secure a designated individual for these specific tasks. This will ensure the course continues to effectively prepare students to engage with diverse communities, understanding the history and systemic factors that may impact their health.

## 6 Acknowledgment of any conceptual, methodological, or material constraints

Our study had several limitations. First, the low survey response rate, as participation was not required in this non-credit bearing course, may have introduced bias into the results. Some students did not complete the entire survey, skipping either the qualitative or quantitative sections. Additionally, the post-survey was developed by an MPH faculty member and has not been previously validated, though the same tool was used for each semester. We also did not evaluate the results of the synchronous Zoom sessions, due to varying topics and presenters.

Another limitation is the lack of a pre-course survey. Without a baseline measure of students’ knowledge or preparedness for fieldwork, we could not assess how the course influenced their understanding. It also made it impossible to determine if or how this course influenced our students’ interactions with the community, or their actions/behaviors during their APE (fieldwork) or engagement with the community in other public health work. Incorporating a brief pre-course self-assessment in future iterations would allow for a clearer examination of perceived learning progression, thereby strengthening the evaluative component. Additionally, administering a post-APE survey, would provide an opportunity for students to reflect on the PSSTC course and identify which content was beneficial to their fieldwork experience.

Finally, comparing data across semesters was challenging because each iteration of PSSTC differed in key aspects, including student cohorts, guest speakers, facilitators, content, and course timing.

## Data Availability

The raw data supporting the conclusions of this article will be made available by the authors, without undue reservation.
